# Hand-assisted laparoscopic Hassab’s procedure for esophagogastric varices with portal hypertension

**DOI:** 10.1186/s40792-017-0387-y

**Published:** 2017-10-23

**Authors:** Takashi Kobayashi, Kohei Miura, Hirosuke Ishikawa, Daiki Soma, Zhengkun Zhang, Takuya Ando, Kizuki Yuza, Yuki Hirose, Tomohiro Katada, Kazuyasu Takizawa, Masayuki Nagahashi, Jun Sakata, Hitoshi Kameyama, Toshifumi Wakai

**Affiliations:** Division of Digestive and General Surgery, Niigata University Graduate School of Medical and Dental Sciences, 1-757, Asahimachi-dori, Chu-o-ku, Niigata, 951-8510 Japan

**Keywords:** Esophageal varices, Gastric varices, Portal hypertension, Hand-assisted laparoscopic surgery, Laparoscopic devascularization, Hassab’s procedure

## Abstract

**Background:**

Laparoscopic surgery for patients with portal hypertension is considered to be contraindicated because of the high risk of massive intraoperative hemorrhaging. However, recent reports have shown hand-assisted laparoscopic surgery for devascularization and splenectomy to be a safe and effective method of treating esophagogastric varices with portal hypertension. The aim of this study is to evaluate the efficacy of hand-assisted laparoscopic devascularization and splenectomy (HALS Hassab’s procedure) for the treatment of esophagogastric varices with portal hypertension.

**Case presentation:**

From 2009 to 2016, seven patients with esophagogastric varices with portal hypertension were treated with hand-assisted laparoscopic devascularization and splenectomy in our institute. Four men and three women with a median age of 61 years (range 35–71) were enrolled in this series. We retrospectively reviewed the medical records for the perioperative variables, postoperative mortality and morbidity, and postoperative outcomes of esophagogastric varices. The median operative time was 455 (range 310–671) min. The median intraoperative blood loss was 695 (range 15–2395) ml. The median weight of removed spleen was 507 (range 242–1835) g. The conversion rate to open surgery was 0%. The median postoperative hospital stay was 21 (range 13–81) days. During a median 21 (range 3–43) months of follow-up, the mortality rate was 0%. Four postoperative complications (massive ascites, enteritis, intra-abdominal abscess, and intestinal ulcer) were observed in two patients. Those complications were treated successfully without re-operation. Esophagogastric varices in all patients disappeared or improved. Bleeding from esophagogastric varices was not observed during the follow-up period.

**Conclusion:**

Although our data are preliminary, hand-assisted laparoscopic devascularization and splenectomy proved an effective procedure for treating esophagogastric varices in patients with portal hypertension.

## Background

Esophagogastric variceal bleeding is a major life-threatening complication of portal hypertension resulting from liver cirrhosis. The 6-week mortality of variceal bleeding is 20% [1]. Most patients can be treated endoscopically [2] or with interventional radiology [3]. Therefore, only some patients require surgery such as esophagogastric devascularization and splenectomy (Hassab’s procedure) [4].

Laparoscopic surgery for patients with portal hypertension is considered to be contraindicated because of the high risk of massive intraoperative hemorrhaging caused by splenomegaly, the existence of collateral vessels around the spleen, low platelet counts, and impaired coagulation factors [5–7]. However, recent cumulative experience and data from laparoscopic surgeries and recent advances in surgical devices, especially vessel sealing systems, have increasingly indicated a laparoscopic approach in certain cases, including patients with liver cirrhosis and portal hypertension [8–10]. Recent reports have shown hand-assisted laparoscopic surgery (HALS) for Hassab’s procedure to be a safe and effective method of treating gastric varices with portal hypertension [11, 12]. We introduced this procedure to our hospital in 2009.

The aim of this study is to evaluate the efficacy of hand-assisted laparoscopic devascularization and splenectomy (HALS Hassab’s procedure) for the treatment of esophagogastric varices with portal hypertension.

## Case presentation

### Indications

HALS Hassab’s procedure was indicated for the treatment of patients diagnosed with liver cirrhosis and esophagogastric varices that were difficult to treat with endoscopic therapy and/or interventional radiology (e.g., balloon-occluded retrograde transvenous obliteration) [3]. The contraindication of HALS Hassab’s procedure was a Child-Pugh grade of C.

### Patients

From May 2009 to December 2016, seven patients with esophagogastric varices with portal hypertension were treated with HALS Hassab’s procedure at our institute. All patients were referred from the Department of Gastroenterology at the same institute. This study reviewed the perioperative laboratory and morphologic data, operative variables, and postoperative outcomes. All values are expressed as the median (range, minimum to maximum).

### Surgical procedures

Before surgery, a laboratory examination was performed routinely. Patients with platelet counts < 5.0 × 10^4^/μl were given a platelet transfusion intra-operatively to reduce blood loss. Pneumococcal vaccination is routinely performed at least 2 weeks before surgery to prevent severe septic complications after splenectomy.

**Fig. 1:**
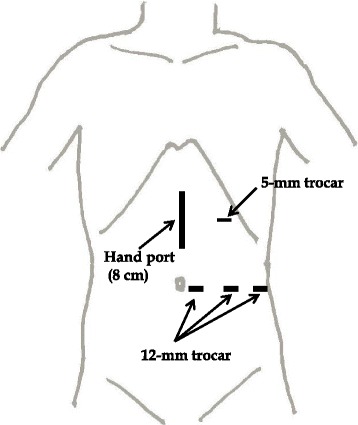
Skin incision and port placement of hand-assisted laparoscopic devascularization and splenectomy. An 8.0-cm upper midline incision was made to introduce a hand port. After the abdomen was insufflated to 10 mmHg with carbon dioxide, the first trocar (12 mm) was inserted on the left side of the umbilicus. A flexible-type laparoscope was placed into the abdomen. Two other 12-mm trocars were inserted under laparoscopic observation to the left of the first trocar and on the left flank of the anterior axillar line, respectively, and one 5-mm trocar was inserted into the left subcostal area

Under general anesthesia, each patient was placed in a semi-decubitus position with the left flank elevated at a 45 ° angle. The operating table was then rotated to render the patient’s position horizontal for skin incision and port insertion. An 8.0-cm upper midline incision was made to introduce a hand port (GelPort; Applied Medical, Rancho Santa Margarita, CA, USA). After the abdomen was insufflated to 10 mmHg with carbon dioxide, the first trocar (12 mm) was inserted on the left side of the umbilicus. A flexible-type laparoscope (LTF Type V3; Olympus, Tokyo, Japan) was placed into the abdomen. Two other 12-mm trocars were inserted under laparoscopic observation to the left of the first trocar and on the left flank of the anterior axillar line, respectively, and one 5-mm trocar was inserted into the left subcostal area (Fig. 1).

After all trocars had been inserted, the patient’s position was returned to the right semi-decubitus position and HALS splenectomy was started. The gastrosplenic ligament was dissected using the LigaSure vessel-sealing system (Covidien, Tokyo, Japan). In most patients, because the gastrosplenic ligament near the upper pole of the spleen was very crowded due to the enlarged spleen and developed collateral vessels, dissection was performed after splenectomy. In cases of enlarged collateral vessels or short gastric veins with a diameter > 7 mm, an endoscopic linear vascular stapler (Endo GIA; Covidien) was used for dissection. We then dissected the splenocolic ligament using a LigaSure vessel-sealing system and mobilized the spleen to visualize the retroperitoneal attachments. After dissection of the retroperitoneal attachments, the upper pole of the spleen was safely dissected away from the diaphragm. Finally, we transected the splenic hilar pedicles with an endoscopic linear vascular stapler, and the spleen was resected. After confirming hemostasis, the spleen was placed into a plastic bag. The resected spleen was cut into two or more pieces in the bag and was carefully removed from the abdomen through the hand port.

Devascularization of the abdominal esophagus and the upper stomach was started from the greater curvature of the upper stomach. Devascularization of the left gastroepiploic and short gastric vessels was performed using the LigaSure vessel-sealing system. The lesser omentum was then opened, and devascularization of the lesser curvature was performed via the same method. The left gastric artery and vein were identified and divided using an endoscopic linear vascular stapler. After the devascularization of the lesser curvature, the vessels around the abdominal esophagus were dissected approximately 5 cm from the esophagogastric junction. The anterior and posterior branches of the vagus nerve were preserved during devascularization of the esophagus. To prevent pyloric stenosis after surgery, pyloric dilatation by finger bougie was routinely performed. After confirmation of hemostasis, a closed type drain was placed to the left subphrenic space routinely.

Enhanced abdominal computed tomography (CT) was routinely performed at 1 week after surgery to evaluate venous thrombosis of the portal venous system. After venous thrombosis was detected by CT, 5000–10,000 units of heparin sodium was immediately started in order to prevent the development of complete portal venous thrombosis. Heparin sodium was converted to warfarin potassium, which was continued until at least 6 months after surgery. The dose of warfarin potassium was controlled by monitoring the prothrombin time international normalized ratio (PT-INR). The target PT-INR ranged from 2.0 to 2.5.

### Evaluation of postoperative complications

The postoperative complications were evaluated as grades I, II, III, IV, or V according to the Clavien-Dindo classification [13]. Grade I complications included any minor deviations from a normal postoperative course without the need for pharmacologic intervention. Grade II complications consisted of complications treated pharmacologically. Grade III complications consisted of complications requiring surgical, endoscopic, or radiologic intervention. Grade IV complications were life-threatening. Grade V complications resulted in patient death. Splenic venous thromboses were not included among postoperative complications in this study. “Early” postoperative complications were defined as complications observed through 30 days after surgery. “Late” postoperative complications were defined as complications observed from 31 days after surgery.

### Evaluation of esophagogastric varices

Esophagogastric varices were evaluated endoscopically based on the published general rules for recording endoscopic findings of esophagogastric varices (second edition) [14]. Endoscopic findings were recorded for two main categories: form (F) and red color signs (RC). The forms of esophagogastric varices were classified into four groups according to shape and size: F0 lesions lack a varicose appearance; F1 lesions are straight, small-caliber varices; F2 lesions are moderately enlarged, beady varices; and F3 lesions are markedly enlarged, nodular, or tumor-shaped varices. Red color signs of esophageal varices were graded as 0, 1, 2, or 3 according to their density and distribution: RC0 = absent; RC1 = small in number and localized; RC2 = intermediate between RC1 and RC3; and RC3 = large in number and circumferential. Red color signs of gastric varices were graded as 0 or 1: RC0 = absent; RC1 = present with red wale markings.

### Outcomes

The patient characteristics in this study are shown in Table 1. The median patient age was 61 years (range, 35–71). Four men and three women were included. The median body mass index was 21.9 kg/m2 (range 19.8–43.0). The etiologies of liver cirrhosis were hepatitis C virus (*n* = 2), recurrent primary sclerosing cholangitis after living donor liver transplantation (*n* = 1), primary biliary cholangitis (*n* = 1), non-alcoholic steatohepatitis (*n* = 1), alcoholic (*n* = 1), and unknown (*n* = 1). One patient had hepatocellular carcinoma The Child-Pugh classification was grade A in three patients and grade B in four. The median Child-Pugh score was 7 (range 6–9).

**Table 1:** Patient characteristics

Case	Age (years)/gender	BMI (kg/m^2^)	Etiology of LC	HCC	Child-Pugh grade (score)
1	35/M	21.9	Recurrent PSC after LDLT	None	B (9)
2	61/F	26.1	PBC	None	B (7)
3	65/F	21.7	HCV	Present	A (6)
4	47/M	43.0	NASH	None	A (6)
5	60/F	21.8	Unknown	None	B (8)
6	71/M	19.8	Alcoholic	None	B (7)
7	63/M	23.7	HCV	None	A (6)

*BMI* body mass index, *LC* liver cirrhosis, *HCC* hepatocellular carcinoma, *M* male, *F* female, *PSC* primary sclerosing cholangitis, *LDLT* living-donor liver transplantation, *PBC* primary biliary cirrhosis, *HCV* hepatitis C virus, *NASH* nonalcoholic steatohepatitis

The operative results are shown in Table 2. The operative time was 455 min (range 310–671), and blood loss was 695 ml (range 15–2395). Four patients (cases 1, 2, 4, and 5) required red blood cell concentrates and/or fresh-frozen plasma transfusion intraoperatively because of preoperative anemia, coagulopathy, and bleeding. Three patients received multiple procedures simultaneously during surgery. Two patients (cases 2 and 5) received laparoscopic cholecystectomy for cholecystolithiasis, and one (case 3) received partial hepatectomy for hepatocellular carcinoma. The weight of the resected spleen was 507 g (range 242–1835). The conversion rate to open surgery was 0% (0/7).

**Table 2:** Operative and postoperative data for seven patients

Case	Operative time (min)	Blood loss (ml)	Additional procedures	Weight of spleen (g)	Conversion to open surgery	Early postoperative complications	Postoperative hospital stay (days)
1	464	1660	None	1835	No	Massive ascites, enteritis	43
2	455	1715	LC	586	No	None	14
3	569	695	Partial hepatectomy	242	No	Intra-abdominal abscess	81
4	671	2395	None	1510	No	None	23
5	423	260	LC	507	No	None	21
6	310	260	None	265	No	None	16
7	311	15	None	404	No	None	13

*LC* laparoscopic cholecystectomy

Early postoperative complications were observed in two patients. One patient (case 1) showed massive ascites and enteritis, which were treated successfully with medication (grade II). Another patient (case 3) showed intra-abdominal abscess, which was treated successfully with interventional radiology (grade III). Portal venous thrombosis was not detected by CT. However, incomplete splenic venous thromboses were detected in all seven patients. Heparin sodium was immediately started in order to prevent the development of complete portal venous thrombosis. No cases showed portal venous thrombosis with anticoagulation therapy. The hospital stay after surgery was 21 days (range 13–81). All seven patients were discharged from our hospital on foot without any sequelae.

Follow-up data were obtained from all seven patients. The follow-up period was 21 months (range 3–43 months). The preoperative and postoperative endoscopic findings of the esophagogastric varices are shown in Table 3. All patients were alive. Regarding the outcomes, esophagogastric varices were improved in 100% (7/7) of cases, and esophageal and gastric varices disappeared in 57% (4/7) and 71% (5/7) of cases, respectively. Bleeding from esophagogastric varices was not observed. One patient (case 1) showed a grade III late complication (bleeding from intestinal ulcer) at 35 months after surgery, which was treated successfully with endoscopic intervention.

**Table 3:** Endoscopic findings and late complications on seven patients

Case	Preoperative endoscopic findings		Postoperative endoscopic findings		Late complications	Follow-up period (months)
	EV	GV	EV	GV		
1	F2, RC2	F2, RC0	None	None	Bleeding from intestinal ulcer	43
2	F1, RC1	F2, RC0	F1, RC0	None	None	40
3	F1, RC0	F2, RC0	None	None	None	31
4	F2, RC1	F2, RC0	F1, RC0	F1, RC0	None	21
5	F3, RC1	F2, RC0	F1, RC0	F1, RC0	None	12
6	F2, RC0	F1, RC0	None	None	None	7
7	F3, RC1	F1, RC0	None	None	None	3

*EV* esophageal varices, *GV* gastric varices

## Discussion

The results of this study indicate that HALS Hassab’s procedure is an effective procedure for treating esophagogastric varices with portal hypertension. However, in three (case 1, 2, and 4) of seven patients, intraoperative blood loss was over 1500 ml, which was greater than the blood loss in previous reports [11, 12, 15, 16]. The operative time was longer in comparison with previous reported data. Possible reasons were included as following: (1) severe upper abdominal adhesion due to the previous living donor liver transplantation (case 1) and severe perigastric adhesion due to the history of multiple gastric ulcers (case 2); and (2) very high body mass index (> 40) (case 4). Laparoscopic splenectomy in patients with severe upper abdominal adhesion or obesity was associated with increased blood loss and longer operative times [17–20]. The patients with the upper abdominal severe adhesion or high body mass index (> 40) should be considered as the contraindication of HALS Hassab’s procedure. Open Hassab’s procedure should be indicated for those patients. Decision for conversion during surgery is also important. The most common reason for conversion to open surgery is uncontrolled bleeding [16]. However, longer operative time or increased blood loss is also a relative indication of conversion even if the bleeding is controlled properly.

Concerning about surgical procedure, the vessel-sealing system and endovascular stapler are very effective devices for dissection and devascularization of collateral vessels and splenic hilum, but the usage of them could not lead to surgical outcome in some cases. Preemptive splenic artery embolization prior to surgery [21] or early splenic artery ligation (arterial trunk in supra-pancreatic area) as in-flow modulation could be promising procedure for safe dissection in huge splenomegaly or massive collateral vessels [22].

Four clinical studies including a total of 28 cases on HALS Hassab’s procedure for esophagogastric varices have been reported [11, 12, 15, 16]. Three of those four studies reported intraoperative blood loss on Hassab’s procedure. Yamamoto et al. reported median intraoperative blood loss was 100 ml (ranged from 50 to 475 ml) in 7 cases [11]. Ando et al. reported median intraoperative blood loss was 640 g (ranged from 24 to 1065 g) in 6 cases [12]. Akahoshi et al. reported intraoperative blood loss was 250 ± 210 ml (mean ± SD) in 10 cases [16]. Although the safety hemorrhage volume during Hassab’s procedure was not reported, WHO guideline for safe surgery required an adequate preparation if there is a risk of more than 500 ml blood loss during surgery [23].

The mortality rate was 0% (0/7), and the morbidity rate (more than Clavien-Dindo Grade II) was 29% (2/7) in the present study. Only one case of in-hospital mortality has been reported [11]. This case died as a result of acute respiratory distress syndrome secondary to aspiration pneumonia. This case of pneumoniae was caused by pyloric stricture after HALS Hassab’s procedure. To minimize the likelihood of this complication, the finger bougie method to dilate the muscle of the pylorus ring is recommended [24, 25]. Reported morbidity rates of HALS Hassab’s procedure range from 30 to 67%. Ando et al. reported three cases of portal thrombus and one case of massive ascites out of six cases [12]. Akahoshi et al. reported 2 cases of postoperative bleeding and 1 case of portal thrombus out of 10 cases [16]. In the present study, the morbidity rate was 29%, and all complications were Clavien-Dindo grade III or less. Fortunately, these complications were treated successfully without re-operation. One study showed a lower morbidity rate with HALS Hassab’s procedure than with open Hassab’s procedure [12]. The reported postoperative hospital stay after HALS Hassab’s procedure ranged from 6.5 to 19.8 days [12, 16]. These lengths were similar to the patients’ postoperative hospital stay (21 days) in our study. Two comparison studies have been performed. One study showed that the postoperative hospital stay was shorter in the HALS Hassab’s group (19.8 days) than in the open Hassab’s group (35.6 days) [12]. The other study also found that the postoperative hospital stay was shorter with HALS Hassab’s procedure than with the open procedure (6.5 vs 15.2 days) [16].

Portal venous thrombosis is frequent complication of splenectomy and can be fatal in patients with liver cirrhosis [26]. The incidence of portal venous thrombosis after splenectomy in cirrhotic patients was from 3 to 78.6% [15, 27]. It is important to determine risk factors for portal venous thrombosis and to prevent portal venous thrombosis after splenectomy in cirrhotic patients. Kawanaka et al. reported decreased antithrombin III (ATIII) activity and large splenic vein diameter (SVD) are risk factors for portal vein thrombosis after splenectomy in liver cirrhosis with portal hypertension [28]. They also reported effective prophylactic protocol for portal venous thrombosis according to the risk level of portal vein thrombosis after splenectomy. Patients at low risk (ATIII activity ≥ 70% and SVD < 10 mm) were not treated; those at high risk (ATIII activity < 70% or SVD ≥ 10 mm) received ATIII concentrates (1500 U/day) for 3 days; and those at highest risk (SVD ≥ 15 mm) received ATIII concentrates for 3 days followed by danaparoid sodium (2500 U/day) for 14 days and warfarin. In the validation cohort, 0 of 14 low-risk and 2 of 32 high-risk patients had portal venous thrombosis. Although 8 of 11 patients at highest risk had temporary portal venous thrombosis, it disappeared within 3 months postoperatively. Finally, only 2 (3.5%) of 57 patients had portal venous thrombosis [28].

The long-term efficacy of HALS Hassab’s procedure for gastroesophageal varices was evaluated. The 5-year non-recurrence rates of esophageal and gastric varices were 100 and 87.5%, respectively. There was no marked difference in the 5-year non-recurrence rate of esophagogastric varices among HALS, open, and pure laparoscopic Hassab’s procedures [16]. In the present study, 100% (7/7) of esophagogastric varices were improved or disappeared, and no bleeding from esophagogastric varices was observed during 21 months of follow-up. Although our follow-up period was relatively short, HALS Hassab’s procedure for esophagogastric varices was deemed effective.

## Conclusions

This study suggested that HALS Hassab’s procedure for esophagogastric varices with portal hypertension was effective for the treatment and prevention of bleeding from esophagogastric varices. There were no mortalities or major complications after HALS Hassab’s procedure during this study period. However, the operative time was longer and blood loss was greater in comparison with previous reported data. Further studies, such as prospective randomized studies, with a greater number of cases are needed to confirm the safety and efficacy of HALS Hassab’s procedure for esophagogastric varices with portal hypertension.

## Abbreviations

ATIII: Antithrombin III
CT: Computed tomography
HALS Hassab’s procedure: Hand-assisted laparoscopic devascularization and splenectomy
HALS: Hand-assisted laparoscopic surgery
PT-INR: Prothrombin time international normalized ratio
SVD: Splenic vein diameter

## References

[1] D’Amico G, De Franchis R, Cooperative Study Group (2003). Upper digestive bleeding in cirrhosis. Post-therapeutic outcome and prognostic indicators. Hepatology.

[2] Stiegmann GV, Goff JS, Michaletz-Onody PA, Korula J, Lieberman D, Saeed ZA, Reveille RM, Sun JH, Lowenstein SR (1992). Endoscopic sclerotherapy as compared with endoscopic ligation for bleeding esophageal varices. N Engl J Med.

[3] Akahoshi T, Hashizume M, Tomikawa M, Kawanaka H, Yamaguchi S, Konishi K, Kinjo N, Maehara Y (2008). Long-term results of balloon-occluded retrograde transvenous obliteration for gastric variceal bleeding and risky gastric varices: a 10-year experience. J Gastroenterol Hepatol.

[4] Hassab MA (1964). Gastroesophageal decongestion and Splenectomy. A method of prevention and treatment of bleeding from esophageal Varices associated with Bilharzial hepatic fibrosis: preliminary report. J Int Coll Surg.

[5] Habermalz B, Sauerland S, Decker G, Delaitre B, Gigot JF, Leandros E, Lechner K, Rhodes M, Silecchia G, Szold A, Targarona E, Torelli P, Neugebauer E (2008). Laparoscopic splenectomy: the clinical practice guidelines of the European Association for Endoscopic Surgery (EAES). Surg Endosc.

[6] Trelles N, Gagner M, Pomp A, Parikh M (2008). Laparoscopic splenectomy for massive splenomegaly: technical aspects of initial ligation of splenic artery and extraction without hand-assisted technique. J Laparoendosc Adv Surg Tech A.

[7] Mahon D, Rhodes M (2003). Laparoscopic splenectomy: size matters. Ann R Coll Surg Engl.

[8] Nicholson IA, Falk GL, Mulligan SC (1998). Laparoscopically assisted massive splenectomy. A preliminary report of the technique of early hilar devascularization. Surg Endosc.

[9] Wang Y, Zhan X, Zhu Y, Xie Z, Zhu J, Ye Z (2010). Laparoscopic splenectomy in portal hypertension: a single-surgeon 13-year experience. Surg Endosc.

[10] Kobayashi T, Miura K, Ishikawa H, Oya H, Sato Y, Minagawa M, Sakata J, Takano K, Takizawa K, Nogami H, Kosugi SI, Wakai T (2014). Laparoscope-assisted Hassab’s operation for esophagogastric varices after living donor liver transplantation: a case report. Transplant Proc.

[11] Yamamoto J, Nagai M, Smith B, Tamaki S, Kubota T, Sasaki K, Ohmori T, Maeda K (2006). Hand-assisted laparoscopic splenectomy and devascularization of the upper stomach in the management of gastric varices. World J Surg.

[12] Ando K, Kurokawa T, Nagata H, Arikawa T, Yasuda A, Ito N, Kotake K, Nonami T (2012). Laparoscopic surgery in the management of hypersplenism and esophagogastric varices: our initial experiences. Surg Innov.

[13] Dindo D, Demartines N, Clavien PA (2004). Classification of surgical complications: a new proposal with evaluation in a cohort of 6336 patients and results of a survey. Ann Surg.

[14] Tajiri T, Yoshida H, Obara K, Onji M, Kage M, Kitano S, Kokudo N, Kokubu S, Sakaida I, Sata M, Tajiri H, Tsukada K, Nonami T, Hashizume M, Hirota S, Murashima N, Moriyasu F, Saigenji K, Makuuchi H, Oho K, Yoshida T, Suzuki H, Hasumi A, Okita K, Futagawa S, Idezuki Y (2010). General rules for recording endoscopic findings of esophagogastric varices (2nd edition). Dig Endosc.

[15] Kakinoki K, Okano K, Suto H, Oshima M, Hagiike M, Usuki H, Deguchi A, Masaki T, Suzuki Y (2013). Hand-assisted laparoscopic splenectomy for thrombocytopenia in patients with cirrhosis. Surg Today.

[16] Akahoshi T, Uehara H, Tomikawa M, Kawanaka H, Hashizume M, Maehara Y (2014). Comparison of open, laparoscopic, and hand-assisted laparoscopic devascularization of the upper stomach and splenectomy for treatment of esophageal and gastric varices: a single-center experience. Asian J Endosc Surg.

[17] Seetahal S, Obirieze A, Cornwell EE, Fullum T, Tran D (2015). Open abdominal surgery: a risk factor for future laparoscopic surgery?. Am J Surg.

[18] Weiss CA, Kavic SM, Adrales GL, Park AE (2005). Laparoscopic splenectomy: what barriers remain?. Surg Innov.

[19] Heniford BT, Park A, Walsh RM, Kercher KW, Matthews BD, Frenette G, Sing RF (2001). Laparoscopic splenectomy in patients with normal-sized spleens versus splenomegaly: does size matter?. Am Surg.

[20] Patel AG, Parker JE, Wallwork B, Kau KB, Donaldson N, Rhodes MR, O'Rourke N, Nathanson L, Fielding G (2003). Massive splenomegaly is associated with significant morbidity after laparoscopic splenectomy. Ann Surg.

[21] Iwase K, Higaki J, Yoon HE, Mikata S, Miyazaki M, Nishitani A, Hori S, Kamiike W (2002). Splenic artery embolization using contour emboli before laparoscopic or laparoscopically assisted splenectomy. Surg Laparosc Endosc Percutan Tech.

[22] Palanivelu C, Jani K, Malladi V, Shetty R, Senthilkumar R, Maheshkumar G (2006). Early ligation of the splenic artery in the leaning spleen approach to laparoscopic splenectomy. J Laparoendosc Adv Surg Tech A.

[23] Haynes AB, Weiser TG, Berry WR, Lipsitz SR, Breizat AH, Dellinger EP, Herbosa T, Joseph S, Kibatala PL, Lapitan MC, Merry AF, Moorthy K, Reznick RK, Taylor B, Gawande AA, Safe Surgery Saves Lives Study Group (2009). A surgical safety checklist to reduce morbidity and mortality in a global population. N Engl J Med.

[24] Hai AA, Singh A, Mittal VK (1986). Closed pyloroduodenal digital dilatation as a complementary drainage procedure to truncal vagotomy. Int Surg.

[25] Yamashita Y, Hirai T, Mukaida H, Yoshimoto A, Kuwahara M, Inoue H, Toge T (1999). Finger bougie method compared with pyloroplasty in the gastric replacement of the esophagus. Surg Today.

[26] Kinjo N, Kawanaka H, Akahoshi T, Tomikawa M, Yamashita N, Konishi K, Tanoue K, Shirabe K, Hashizume M, Maehara Y (2010). Risk factors for portal venous thrombosis after splenectomy in patients with cirrhosis and portal hypertension. Br J Surg.

[27] Kawanaka H, Akahoshi T, Kinjo N, Konishi K, Yoshida D, Anegawa G, Yamaguchi S, Uehara H, Hashimoto N, Tsutsumi N, Tomikawa M, Maehara Y (2010). Impact of antithrombin III concentrates on portal vein thrombosis after splenectomy in patients with liver cirrhosis and hypersplenism. Ann Surg.

[28] Kawanaka H, Akahoshi T, Itoh S, Iguchi T, Harimoto N, Uchiyama H, Yoshizumi T, Shirabe K, Takenaka K, Maehara Y (2014). Optimizing risk stratification in portal vein thrombosis after splenectomy and its primary prophylaxis with antithrombin III concentrates and danaparoid sodium in liver cirrhosis with portal hypertension. J Am Coll Surg.

